# Enhanced mucosal contractile activity following rapid, reversible inhibition of bladder activity by dimethyl sulfoxide (DMSO)

**DOI:** 10.1007/s00345-026-06806-2

**Published:** 2026-09-21

**Authors:** L. Katugampola, E. Falk, C. Moro, D. J. Sellers, C. R. Chapple, R. Chess-Williams

**Affiliations:** 1 Department of Urology, Royal Hallamshire Hospital, Sheffield, UK; 2 Centre for Urology Research, Faculty of Health Sciences & Medicine, Bond University, Queensland, 4229 Australia

**Keywords:** Urinary bladder, Bladder mucosa, Detrusor, Dimethyl sulfoxide (DMSO), Human bladder, Urothelium

## Abstract

**Purpose:**

Intravesical dimethyl sulfoxide (DMSO) is used to treat interstitial cystitis/bladder pain syndrome, but its mechanisms of action are not fully understood. The study aim was to investigate the effects of DMSO on contractility of the bladder detrusor and the mucosa (urothelium/lamina propria).

**Methods:**

Contractile responses of isolated detrusor muscle from pig and human bladder were observed in the absence and presence of DMSO (1.7–25%), and after 4 h of washing. The effects of DMSO and recovery were also examined in strips of pig bladder mucosa (urothelium/lamina propria).

**Results:**

In detrusor strips from pig and human, DMSO inhibited contractions to carbachol and at the highest concentration (25%) responses were completely abolished in all tissues. However, after a 4-hour washing period, detrusor responses to carbachol recovered completely. Isolated mucosal strips spontaneously developed phasic contractile activity that was completely abolished within 10 min of DMSO (25%) exposure. Basal tension was also reduced by 49.1 ± 9.4%. The effects of DMSO on the mucosa were rapidly reversed with washing. Contractile activity was completely recovered to initial levels within 12.6 ± 2.9 min and was enhanced above initial levels by 24 ± 2 min. Responses to carbachol, isoprenaline and neurogenic responses were completely abolished by DMSO (25%), but responses recovered completely following washing and carbachol responses significantly enhanced.

**Conclusion:**

These results demonstrate the powerful, but reversible, effects of DMSO on detrusor and mucosal contractile activity. The enhanced mucosal contractile activity upon washout may explain the flare up of symptoms observed in some patients following treatment.

## Introduction

Interstitial cystitis/bladder pain syndrome (IC/BPS) is a chronic, often debilitating condition characterized by chronic pelvic pain or discomfort, urinary urgency and urinary frequency in the absence of identifiable infection or other pathology [1]. The condition is multifactorial with etiologies ranging from inflammatory phenotypes demonstrating epithelial denudation (Hunner type), to patients lacking bladder inflammation, but with urothelial, muscle or neurophysiological dysfunction [2, 3]. Epidemiological estimates vary due to differing diagnostic criteria, but prevalence is reported at 2.7% to 6.5% in women and slightly lower in men [4, 5].

There are no curative treatments for IC/BPS and non-surgical options for symptom control are limited. Dimethyl sulfoxide (DMSO), approved for intravesical administration for IC in 1978, remains the only FDA‑approved intravesical therapy for IC/BPS. Intravesical delivery enables high local concentrations while limiting systemic effects [6], and has demonstrated benefits in patients with Hunner lesions, improving voiding parameters and reducing pelvic pain [7–10]. A recent systematic review and meta-analysis confirmed intravesical DMSO as a safe and effective treatment [11].

Clinical response can however be variable, ranging from 25% to 70%, and symptom relief is often incomplete or temporary [2]. Adverse effects include bladder irritation, discomfort during instillation, and a characteristic garlic‑like odour due to systemic absorption [2, 12]. DMSO is sometimes administered with other agents such as local anaesthetics, corticosteroids or barrier agents, but combinations do not appear to enhance therapeutic benefit over DMSO alone [13]. These limitations contribute to inconsistent patient adherence and emphasise the need for a deeper understanding of its mechanisms of action within the bladder wall.

Reported actions contributing to DMSO’s beneficial effects may include anti‑inflammatory [14, 15], muscle relaxation, collagen inhibition and direct effects on afferent nerves [12, 16, 17], but few have examined the effects of DMSO on bladder contractility. The tissue layers that have the greatest exposure to DMSO during intravesical treatment are the urothelium and lamina propria, which have several roles in bladder function, releasing mediators such as ATP and acetylcholine, but also possess contractile properties [18] and generate tonic contractions to muscarinic agonists and spontaneous phasic contractile activity [19, 20]. Despite DMSO having direct contact with this tissue layer, its effects on its contractile function are largely unknown. The aims of the present study were therefore to identify the effects of DMSO on strips of porcine detrusor and urothelium/lamina propria. The reversibility and functional recovery of tissues from DMSO was also examined.

## Methods

Fresh bladders from Large White-Landrace pigs (6 months old, ~ 80 kg) were obtained from a local abattoir and immersed in cold (4 °C) Krebs-bicarbonate solution (composition in mM: NaCl 188.4, NaHCO_3_ 24.9, CaCl_2_ 1.9, MgSO_4_ 1.15, KCl 4.7, KH_2_PO_4_ 1.15 and D-glucose 11.7). Human detrusor muscle from the bladder dome were obtained from patients undergoing cystectomy (cancer of the bladder neck) with approval of South Sheffield Ethics Committee (UK) and informed consent. Tissues were transported in Krebs-bicarbonate solution. Porcine bladders were opened longitudinally and strips of the anterior dome removed. One or two strips were taken from each human or porcine bladder sample respectively, each strip used for only one experimental protocol, thus n (strips) is equivalent to N (animals).

Detrusor strips (10 mm x 3 mm, mucosa and serosa removed) and mucosal strips (urothelium/lamina propria) were mounted in tissue baths containing Krebs-bicarbonate solution (37 °C, 95%O_2_/5%CO_2_). Tissues were equilibrated (60 min), washed every 10 min and resting tensions adjusted to 1 g. Isometric tension was measured via UF1 force transducers, a PC and Cambridge Electronic Design interface using “CHART” software.

Following equilibration, cumulative concentration-response curves to carbachol or potassium chloride (KCl) were obtained in detrusor strips. Tissues were washed for 45 min, incubated with DMSO (1.7%, 3.3%, or 25%, 30 min), and a second concentration-response curve to carbachol or KCl constructed. Additional experiments using pig detrusor examined recovery of responses following 25% DMSO (30 min), with tissues washed for 4 h before second concentration-response curves were constructed. Time-matched control experiments without DMSO accounted for time-dependent changes.

Mucosal strips were equilibrated (60 min) and developed spontaneous phasic contractile activity. The effect of DMSO (1.7, 3.3 or 25%) on spontaneous phasic activity was investigated. After 30 min exposure to 25% DMSO tissues were washed repeatedly and spontaneous activity allowed to recover. In separate strips, responses to the muscarinic agonist carbachol, β-adrenoceptor agonist isoprenaline or electrical field stimulation (EFS) (50 V, 0.5ms pulse width, 5 s trains every 100 s) were measured in the absence of DMSO, the presence of 25% DMSO and following washout and recovery of spontaneous activity.

### Drugs and solutions

Carbamylcholine chloride (carbachol), isoprenaline hydrochloride, KCl, DMSO were obtained from Sigma (St Louis, USA). Solutions were freshly prepared in distilled water and diluted in Krebs-bicarbonate solution.

### Statistical analysis

Responses to carbachol or KCl were expressed as percentage of maximal response of the first concentration-response curve in each experiment. EC50 values (molar concentration producing half-maximal response) were obtained from non-linear regression analysis using GraphPad Prism^®^ and mean pEC50 (-logEC50) with SEM calculated. For mucosal strips, frequency and amplitude of spontaneous contractions and baseline tension were measured. Paired two-tailed Student’s *t*-tests were used for comparisons.

## Results

### Effects of DMSO on detrusor contractile activity

**Fig. 1:**
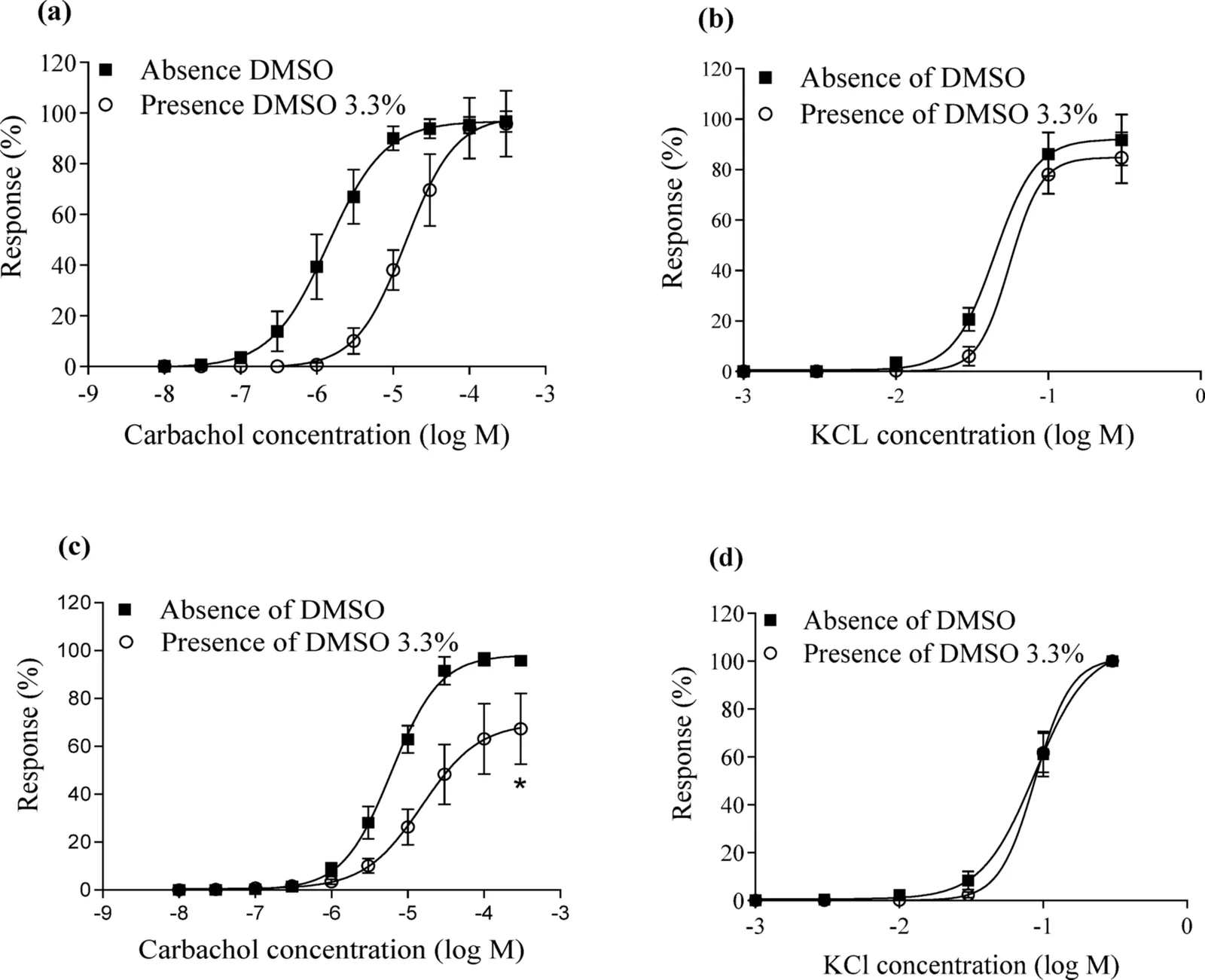
Effect of DMSO (3.3%) on responses of human (a and b) and pig (c & d) detrusor muscle to carbachol and potassium chloride. Responses are expressed as a percentage of the control curve (absence of DMSO) maximum contraction. Data is mean ± SEM, *n* = 4–5. * *P* < 0.05 versus responses in the absence of DMSO, paired two-tailed Student’s *t*-test

Carbachol and KCl produced concentration-dependant contractions of human and pig detrusor (Fig. 1). DMSO (1.7% and 3.3%) produced significant (*P* > 0.05) rightward shifts of carbachol concentration-response curves in both (Fig. 1; Table 1) and depressed maximum responses significantly in pig detrusor (Table 1). In contrast these concentrations did not significantly affect KCl responses (Fig. 1; Table 1).

**Fig. 2:**
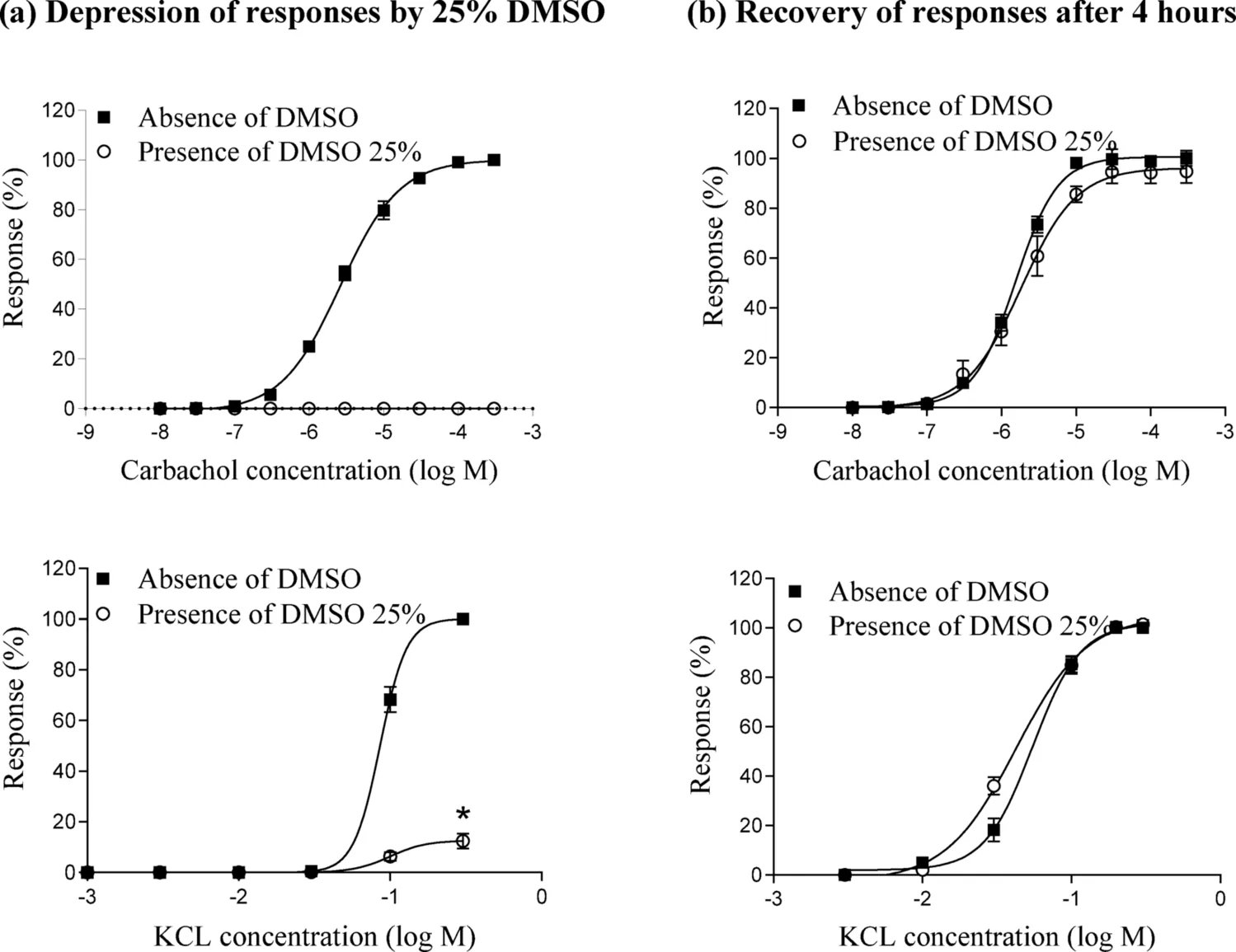
Effect of DMSO (25%) on contractile responses of pig detrusor muscle to carbachol and potassium chloride following 30-minute incubation (a) and recovery of responses following 4-hour washout (b). Responses are expressed as a percentage of the control curve (absence of DMSO) maximum contraction. Data is mean ± SEM, (*n* = 6). * *P* < 0.05 versus responses in the absence of DMSO, paired two-tailed Student’s *t*-test

With 25% DMSO, carbachol-induced contractions were completely abolished in pig detrusor, even at high concentrations (1mM) (Fig. 2). Maximal responses to KCl were also greatly reduced (> 85%) by 25% DMSO (Fig. 2), from 13.61 ± 1.30 g to 1.59 ± 0.35 g (*P* < 0.05), without altering pEC50 values (0.98 ± 0.08 to 0.96 ± 0.03).

### Recovery of detrusor contractile activity after DMSO treatment

After 30 min with 25% DMSO and 4-hour washout, pig detrusor responses to carbachol and KCL were completely recovered (Fig. 2). Maximum responses to carbachol were similar before DMSO (16.02 ± 3.62 g) and after recovery (20.11 ± 4.26 g), and for KCl (control 19.24 ± 2.35 g vs. recovered 19.51 ± 2.41 g).

### Effects of DMSO on mucosa (urothelium/LP) contractile activity

**Fig. 3:**
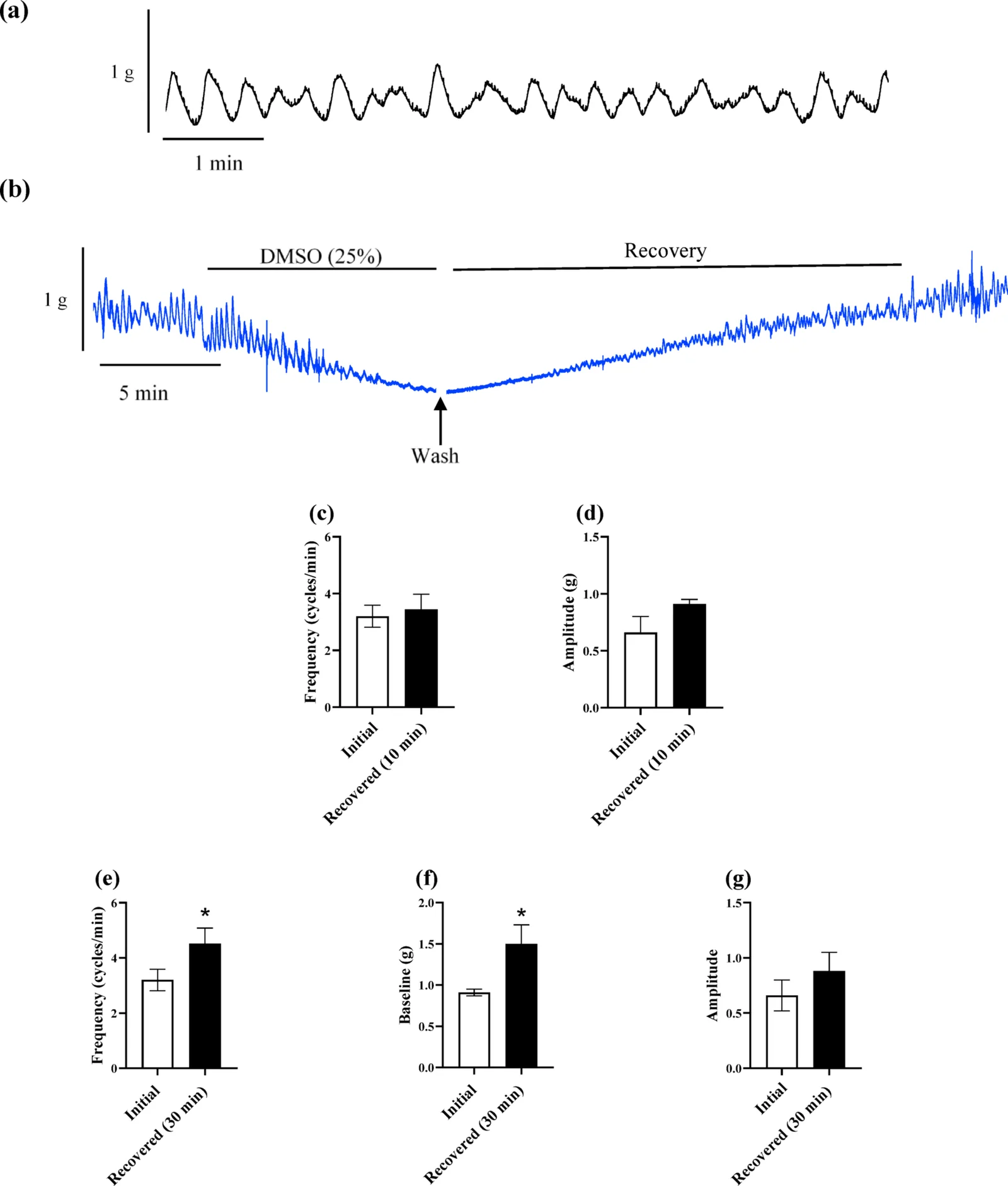
Typical experimental trace depicting normal spontaneous phasic contractile activity of mucosal strips (urothelium/LP) (a). Typical effect of DMSO (25%) on spontaneous phasic contractile activity and recovery, followed by increase, of activity following washout (b)

Pig mucosa developed spontaneous phasic contractile activity (frequency 3.16 ± 0.23 cycles/min; amplitude 0.58 ± 0.10 g, *n* = 13). DMSO (1.7% and 3.3%) slightly decreased basal tension but did not affect frequency or amplitude. However, DMSO (25%) completely abolished spontaneous phasic contractile activity within 9 min and reduced basal tension by 49.1 ± 9.4% (*n* = 5, Fig. 3).

### Recovery of mucosal phasic spontaneous contractile activity after DMSO treatment

Following 30 min with DMSO (25%), tissues were washed and contractile activity recovered quicky. Basal tension returned to baseline by 12.6 ± 2.9 min (Fig. 3) and recovered completely, as did phasic frequency and amplitude (Fig. 3). Interestingly, following washout, contractile activity continued to improve, even beyond the initial activity before addition of DMSO, increasing with washing but then plateauing by 24 ± 2 min. All three parameters of contractile activity were elevated compared to initial values (Fig. 3). Baseline tension was increased by 64.8%, frequency and amplitude by 41.3% and 33.3% respectively, and increases for baseline and frequency were statistically significant (*p* = 0.022 and 0.044 respectively).

**Fig. 4:**
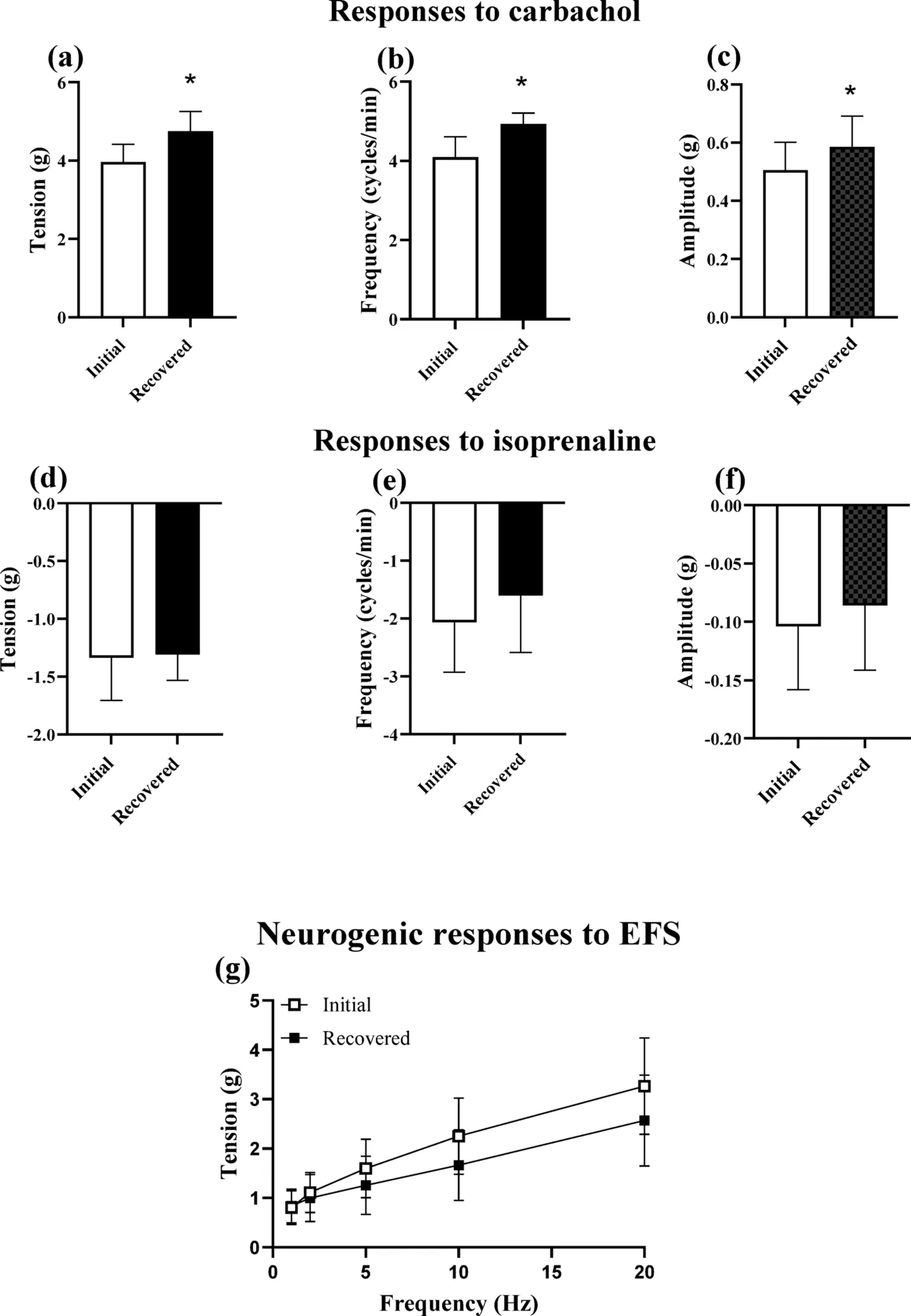
Responses of mucosal strips (urothelium/LP) to carbachol (10µM), isoprenaline (10µM) and electrical field stimulation (1–20 Hz). Data is the change in baseline tension (a, c, g), and frequency (b & d) and amplitude (c & f) of spontaneous activity pre-DMSO (25%) (initial response) versus response at 30 min following washout of DMSO. Data is mean ± SEM, (*n* = 5–7). * *P* < 0.05 vs. initial response, paired two-tailed Student’s *t*-test

In separate experiments, whilst 3.3% DMSO did not affect mucosal contractile responses to carbachol (10µM), relaxation responses to isoprenaline or neurogenic response to EFS, all responses were completely abolished in the presence of 25% DMSO (Fig. 4). Following washout of DMSO, responses to EFS and isoprenaline were restored to initial levels, whereas those to carbachol were significantly elevated compared to initial values (Fig. 4),

**Table 1:** Effects of DMSO on human and pig detrusor muscle responses to carbachol and potassium chloride

% DMSO		pEC_50_			Maximum responses (g)		
		Absence of DMSO	Presence of DMSO	SHIFT	Absence of DMSO	Presence of DMSO	% DEPRESSION
Human							
Carbachol	1.7%	5.23 ± 0.19	5.09 ± 0.28	1.38 *	8.41 ± 2.59	7.97 ± 2.21	5.23%
	3.3%	5.93 ± 0.22	4.83 ± 0.09	12.6 *	4.99 ± 1.18	4.87 ± 1.51	2.41%
KCl	1.7%	1.35 ± 0.36	1.20 ± 0.13	1.4	5.40 ± 1.23	5.52 ± 1.14	0.00%
	3.3%	1.27 ± 0.11	1.30 ± 0.01	0.93	5.43 ± 0.41	4.65 ± 0.86	14.4%
Pig							
Carbachol	1.7%	5.42 ± 0.07	5.06 ± 0.04	2.3 *	19.88 ± 4.10	15.21 ± 3.79	23.5% *
	3.3%	5.41 ± 0.10	4.74 ± 0.11	4.7 *	16.64 ± 1.54	11.16 ± 2.69	32.9% *
	25%	5.59 ± 0.05	-	-	17.13 ± 0.05	0	100% *
KCl	1.7%	1.04 ± 0.06	1.16 ± 0.07	0.75	13.04 ± 2.02	14.6 ± 2.52	0.00%
	3.3%	1.1 ± 0.04	1.05 ± 0.07	1.12	21.5 ± 1.89	21.17 ± 2.1	2.00%
	25%	0.98 ± 0.08	0.96 ± 0.03	1.05	13.61 ± 1.30	1.59 ± 0.35	88.32% *

Mean (± SEM) pEC_50_ values and maximum responses. SHIFT indicates the fold change in pEC_50_ between responses in the absence and presence of DMSO. * *P* < 0.05 versus absence of DMSO, paired two tailed Student’s *t*-test (*n* = 4 or greater)

Frequency and amplitude data showing recovery of spontaneous phasic activity of mucosal strips (urothelium/LP) following washout of DMSO (25%) (c & d). Data shows initial (pre-DMSO) spontaneous activity and recovered spontaneous activity (post-DMSO) following 10 min washout. Data is mean ± SEM, (*n* = 5).

Frequency and amplitude data showing increase in spontaneous phasic activity of mucosal strips (urothelium/LP) beyond the initial (pre-DMSO) levels following washout of DMSO (25%) (e, f & g). Data shows the increased activity at 30 min. Data is mean ± SEM, (*n* = 5). * *P* < 0.05 versus initial level in the absence of DMSO, paired two-tailed Student’s *t*-test.

## Discussion

IC/BPS is a multifactorial condition covering an unknown number of phenotypes. Clinically, patients may present with inflammation and mucosal lesions (Hunners) or with functional etiologies involving the urothelium, detrusor muscle and/or peripheral and central neural pathways [21]. This variability in pathological mechanisms makes IC/BPS difficult to characterise in individual patients and challenging to treat.

DMSO has recognised benefit in IC patients with Hunner lesions [7–10], yet its mechanism of action remains unclear. Experimentally, it exerts anti-inflammatory actions [14, 15], increases epithelial permeability [22], relaxes detrusor muscle [23], stimulates bladder afferent neurons, decreases bladder capacity and causes release of nitric oxide from dorsal root ganglia and bladder strips [17]. In a normal porcine urothelium-intact in vitro model DMSO reduced mucosal thickness and evoked high ATP release [24].

Since detrusor smooth muscle is the primary contractile element of the bladder wall, any drug depressing detrusor contraction may be detrimental to voiding, while a drug enhancing detrusor sensitivity may cause overactive bladder symptoms. In the pig bladder, low concentrations of DMSO (1.7 and 3.3%) depressed contractile responses to the muscarinic agonist carbachol, causing rightward shifts of concentration-response curves. To determine whether this effect was selective for muscarinic responses or represented a general depressant effect, responses to KCl were examined. Unlike muscarinic receptor-mediated stimulation, where contraction is mediated via the intracellular messengers inositol triphosphate and diacylglycerol [25], KCl causes influx of extracellular calcium and direct activation of contractile mechanisms independent of second messenger systems. Thus, KCl-induced contractions reflect general tissue responsiveness, rather than specific receptor-mediated mechanisms. Surprisingly, low concentrations of DMSO caused rightward shifts of the carbachol concentration-response curves without altering responsiveness to KCl. To our knowledge, this is the first report of a specific effect of DMSO on muscarinic receptor-mediated responses in pig and human detrusor. Since DMSO is widely used at low concentrations as a solvent for dissolving drugs in pharmacological research, these results highlight the need for caution when using DMSO in this manner, particularly when examining muscarinic receptor-mediated responses. Clinically, much higher concentrations of DMSO are employed intravesically, but the low concentrations used in this study help define the threshold concentrations required for inhibitory actions.

A small number of human tissues were available to confirm that inhibitory effects of DMSO seen in porcine tissues were representative of its actions in human detrusor. As in porcine tissues, DMSO depressed human detrusor contraction to carbachol without affecting KCl responses. In human detrusor 3.3% DMSO caused a rightward shift of carbachol curves rather than depressing maximum responses. This may suggest a greater functional reserve within the muscarinic pathway in human detrusor, depression of responses possibly causing a rightward shift, with depression of maximum responses only at high concentrations. This is supported by the lower pEC50 for carbachol in human tissues, indicating that human detrusor was more sensitive to muscarinic stimulation than pig. However, in both species the overall effect was similar, low concentrations of DMSO depressing muscarinic responses without altering potassium responses.

The effects of a higher concentrations of DMSO were also examined. During intravesical treatment of patients for IC/BPS, concentrations up to 50% for up to 30 min are used [9]. The exact concentration in the bladder lumen will vary due to dilution by urine from the kidneys. We used 25% DMSO, well below typical clinical concentrations, yet the effects were unexpectedly extreme, with complete abolition of detrusor responses to both carbachol and KCl. Even more surprising was the degree of recovery after a 4-hour washout, with complete recovery of responses to control levels for both carbachol and KCl. Thus, even at concentrations well below those used in intravesical treatments, DMSO completely abolishes all detrusor contraction while in contact with the tissue, but tissues recovery quickly following removal. Powerful depressant effects on detrusor smooth muscle were seen in rodents and rabbits [26, 27], potentially due to inhibition of myosin light chain (MLC) phosphorylation [28]. DMSO can activate tyrosine kinases [29], which in turn influence MLC kinases, and could explain the observed non-specific depressant actions [28].

When administered systemically DMSO exerts several side effects, most commonly gastrointestinal [30]. For IC/BPS, DMSO is administered intravesically to prevent systemic effects. Thus, the tissue most exposed to DMSO is the mucosa, the urothelium and underlying lamina propria. Once considered a simple protective barrier, the urothelium is now known to play critical roles in regulating detrusor function and sensory signalling [31, 32]. Furthermore, it possesses contractile properties, probably related to the thin layer of smooth muscle (muscularis mucosa) within the lamina propria [18–20, 33]. Mucosal strips develop spontaneous contractile activity, the function of which is not fully understood, but may contribute to mucosal folding during bladder emptying or sensitisation of suburothelial sensory nerves. In a feline cystitis model, waves of activity originated in the mucosa and spread into the detrusor layers, possibly contributing to overactive symptoms [34]. There have, to our knowledge, been no reports of the effect of DMSO on mucosal spontaneous contractile activity, or recovery responses following treatment. We found that, as with the detrusor, low concentrations of DMSO mildly inhibited mucosal contractions, while 25% DMSO completely abolished phasic contractions, agonist-induced and neurogenic responses, simultaneously relaxing tissues. As in detrusor, mucosal responses recovered rapidly upon washout.

What is clear from these studies is that DMSO has alarge depressant effect on all currently examined contractile activity, abolishing detrusor contraction and mucosal phasic contractions and causing mucosal relaxation. The other major, very clear finding, is that these inhibitory effects can recover rapidly and completely. Thus, while in contact with the bladder wall DMSO is likely depressing bladder contractions, but these actions are unlikely to contribute to its clinical efficacy, since it will be quickly removed from the bladder lumen by urine and the bladder wall by normal blood flow.

One of the most unexpected results is that after treating mucosal tissues with DMSO the spontaneous and carbachol-induced contractile responses were enhanced following its removal, with contractile activity recovered to pre-DMSO levels in less than 10 min, establishing its rapid washout from tissues. However, the spontaneous contractile activity continued to improve and stabilize at a new higher level by about 25 min, and carbachol-induced responses were also elevated at this point. This phenomenon has not previously been reported in experimental mechanistic studies, but may be related to clinical observations where patients often experience an initial flare up of symptoms after intravesical DMSO treatment, which normally subside within 72 h [35] but may last up to 2 weeks [9].

The mechanism involved in the enhanced contractile activity is unclear, but mucosal contractile activity is also enhanced during tissue stretch [19] and is the result of M3 muscarinic receptor stimulation by acetylcholine released from the urothelium [20]. Enhanced urothelial acetylcholine release is unlikely in the current situation since our group showed that direct application of DMSO to the mucosal surface in a modified Ussing chamber, causes a large initial urothelial release of acetylcholine, but this results in a depressed subsequent release due to acetylcholine depletion [24]. An alternative explanation for excessive M3 receptor activation could be elevated acetylcholine levels. In this respect, it is interesting that DMSO can inhibit acetylcholinesterase, reducing the breakdown and elevating levels of acetylcholine [28]. This mechanism may also explain the enhanced nerve-evoked detrusor responses reported following DMSO treatment in vitro [24]. However, it must be noted that in this previous study, detrusor responses to carbachol were not investigated during DMSO treatment, but were obtained after luminal DMSO treatment. The rapid recovery of detrusor strips in the current study, now explains the lack of effects on detrusor responses observed in that previous study examining luminal DMSO pretreatment.

How an increase in mucosal contractile activity post-DMSO may affect afferent nerve activity is unclear, but spontaneous micro-contractions of the bladder wall have been shown to cause spikes in afferent nerve activity, indicating the close relationship between the two [36]. Since afferent nerves are found in the lamina propria, they would be particularly sensitive to any contractile activity in this region of the bladder wall. As this phenomenon was only observed after removal of DMSO, it may explain the reports of symptom flare up following treatment [9, 35].

## Conclusion

DMSO at low concentrations (1–3%) depresses detrusor cholinergic contraction without affecting overall contractile function, and at higher concentrations (above 25%) completely abolishes all contractile activity in the mucosa and the detrusor. During intravesical treatment of patients (up to 50% DMSO) these effects are likely to occur, but are unlikely to be persistent due to the very rapid recovery of tissues. However, washout of DMSO from the mucosa after treatment enhances phasic contractile activity and muscarinic receptor-mediated contractility, and this phenomenon may possibly explain the flare up of symptoms experienced by some patients after treatment.

## Data Availability

All data supporting the findings of this study are included within this paper and the figure files.
